# Position statement on the role of healthcare professionals, patient organizations and industry in European Reference Networks

**DOI:** 10.1186/s13023-016-0383-5

**Published:** 2016-01-25

**Authors:** Carla E. M. Hollak, Marieke Biegstraaten, Matthias R. Baumgartner, Nadia Belmatoug, Bruno Bembi, Annet Bosch, Martijn Brouwers, Hanka Dekker, Dries Dobbelaere, Marc Engelen, Marike C. Groenendijk, Robin Lachmann, Janneke G. Langendonk, Mirjam Langeveld, Gabor Linthorst, Eva Morava, Bwee Tien Poll-The, Shamima Rahman, M. Estela Rubio-Gozalbo, Ute Spiekerkoetter, Eileen Treacy, Ronald Wanders, Johannes Zschocke, Rob Hagendijk

**Affiliations:** Department of Internal Medicine, Division of Endocrinology and Metabolism, SPHINX, Amsterdam Lysosome Center, Academic Medical Center, Amsterdam, The Netherlands; Division of Metabolism & Children’s Research Center, University Children’s Hospital Zürich, Zürich, Switzerland; Department of Internal Medicine, Referral Center for Lysosomal Diseases, Hôpitaux Universitaires Paris Nord Val-de-Seine, Beaujon Assistance Publique-Hôpitaux de Paris, Clichy, France; Centre for Rare Diseases, Academic Medical Centre Hospital “Santa Maria della Misericordia”, Udine, Italy; Department of Pediatric Metabolic Disorders, Academic Medical Center, Amsterdam, Netherlands; Department of Internal Medicine, Division of Endocrinology and Metabolic Diseases, Maastricht University Medical Centre, Maastricht, The Netherlands; VKS: Dutch Patient Organization for Metabolic Diseases, Zwolle, The Netherlands; Medical Reference Center for Inherited Metabolic Diseases, Jeanne de Flandre University Hospital and RADEME Research Team for Rare Metabolic and Developmental Diseases, EA 7364 CHRU Lille, 59037 Lille, France; Department of Neurology and Department of Pediatric Neurology, Academic Medical Center, Amsterdam, The Netherlands; Charles Dent Metabolic Unit, National Hospital for Neurology and Neurosurgery, University College London Hospitals, London, UK; Department of Internal Medicine, Netherlands Porphyria Center, Center for Lysosomal and Metabolic Diseases, Erasmus MC, Rotterdam, The Netherlands; Children’s Hospital Leuven, University of Leuven, Leuven, Belgium; Mitochondrial Research Group, Genetics and Genomic Medicine, UCL Institute of Child Health and Metabolic Unit, Great Ormond Street Hospital, London, UK; Departments of Paediatrics and Laboratory Genetic Metabolic Diseases, Maastricht University Hospital, Maastricht, The Netherlands; Department of Pediatrics, Adolescent Medicine and Neonatology, University Children’s Hospital, Freiburg, Germany; National Centre for Inherited Metabolic Disorders, Children’s University and Mater University Hospitals, Dublin, Ireland; Department of Genetic Metabolic Diseases, SPHINX, Amsterdam Lysosome Center, Academic Medical Center, Amsterdam, The Netherlands; Division of Human Genetics, Medical University Innsbruck, Innsbruck, Austria; Amsterdam Institute for Social Science Research, University of Amsterdam, Amsterdam, The Netherlands; Founder and Chair Metabolic Power Foundation, a fund raising charity for more awareness and research into metabolic diseases, Haarlem, The Netherlands

**Keywords:** European Reference Network, Rare diseases, Orphan diseases, Industry, Conflict of interest

## Abstract

A call from the EU for the set-up of European Reference Networks (ERNs) is expected to be launched in the first quarter of 2016. ERNs are intended to improve the care for patients with low prevalent or rare diseases throughout the EU by, among other things, facilitating the pooling and exchange of experience and knowledge and the development of protocols and guidelines. In the past, for example where costly orphan drugs have been concerned, industry has played an important role in facilitating consensus meetings and publication of guidelines. The ERNs should provide a unique opportunity for healthcare professionals and patients to lead these activities in an independent way. However, currently costs for networking activities are not to be covered by EU funds and alternative sources of funding are being explored. There is growing concern that any involvement of the industry in the funding of ERNs and their core activities may create a risk of undue influence. To date, the European Commission has not been explicit in how industry will be engaged in ERNs. We believe that public funding and a conflict of interest policy are needed at the level of the ERNs, Centers of Expertise (CEs), healthcare professionals and patient organizations with the aim of maintaining scientific integrity and independence. Specific attention is needed where it concerns the development of clinical practice guidelines. A proposal for a conflict of interest policy is presented, which may support the development of a framework to facilitate collaboration, safeguard professional integrity and to establish and maintain public acceptability and trust among patients, their organizations and the general public.

## Background

The launch of European Reference Networks (ERNs) for rare diseases should facilitate collaborations between centres of expertise (CEs) and improve the quality of patient care in EU member states and beyond. The call for the actual establishment of these networks is expected to be published in early 2016. At the second conference on ERNs in Lisbon [1], it was clear that there is no specific guidance from the European Commission (EC) on the interaction of care providers with the industry in either CEs or ERNs. In addition, at that time the EC had no plans to provide any funding for these networks. Whether there will be any financial support for ERNs at the level of member states is also unclear. This is unfortunate. The launch of ERNs creates an opportunity for healthcare professionals and patient organizations to work together to improve the quality of care for patients all over Europe, but for this to be effective, an infrastructure will be required. This will need financial support, which must be demonstrable independent of private interest.

The, mainly pharmaceutical, industry currently funds patient care, third party research and several of the activities that will become part of the core business of the ERN. Examples of such support are the set-up of care pathways, consensus guidelines and awareness studies in addition to the support of continuing medical education (CME). While these initiatives have sometimes led to valuable contributions to patient care, they have also raised concerns about the increasing participation and prominent role of the industry in programs that should be the sole responsibility of healthcare professionals [2, 3]. Going forward, to avoid any suggestion of an inappropriate mingling of private and public health interests, industry should not be directly involved in the funding or initial set-up of ERNs. Current guidance contained in EU law and the European Federation of Pharmaceutical Industries and Associations (EFPIA) code of conduct may not prove sufficient and we believe that more focused regulations and public funds are needed to guarantee the independence and scientific integrity of networks, member institutions and their collaborators.

### European Reference Networks

In directive 2011/24/EU (Directive on the application of patients’ rights in cross-border healthcare, [4]) the EC commits itself and the EU member states to “support the development of European Reference Networks between healthcare providers and Centres of Expertise in Member States, in particular in the area of rare diseases” (Art. 12 and 13). These European Reference Networks (ERNs) are intended to support care for patients with low prevalent or rare diseases (RD) throughout the EU, and should provide the framework for healthcare pathways for RD patients through a high level of integrated expertise.

The European Union Committee of Experts in Rare Diseases (EUCERD), replaced by the Commission Expert Group on Rare Diseases, has developed recommendations on quality criteria for centres of expertise for RD as well as for specific needs for RD ERNs [5–7]. The core content of these recommendations is in agreement with the relevant sections of the EC Delegated and Implementing Acts [8, 9]. These Acts provide the binding, legal criteria and conditions that ERNs and their healthcare providers should fulfil, as well as the criteria for establishing and evaluating ERNs and their members. In summary, nationally designated centres of expertise (CEs) are the core participants in RD ERNs, but different forms of affiliation to an RD ERN (association, collaboration) are allowed for other healthcare providers in smaller countries. The main purpose of these networks is to improve clinical care for RD patients by, among other things, facilitating the pooling and exchange of experience and knowledge and the development of protocols and guidelines in order to ensure equal access to accurate information, appropriate and timely diagnosis and highly specialised and high quality care for RD patients. At a later stage, networks can apply for EU grants, which may benefit RD research as well. The Commission Expert Group on Rare Diseases has recommended to group rare diseases in thematic networks [7]. An EU call for the set-up of ERNs is expected for the first quarter of 2016.

Although the Acts are clear in how ERNs are to be set up and evaluated, they are not explicit in how industry can be engaged in ERNs. We believe that to clarify this issue for the various ERNs is of key importance to ensure and guarantee the independence of healthcare providers and the scientific integrity of medical research is fully guaranteed while allowing for the limited participation of industry in the work of ERNs where appropriate. Combining the work of ERNs and CEs with industry while maintaining professional autonomy and integrity as well as public acceptability and trustworthiness is a challenge. In the field of inborn errors of metabolism, a group of rare disorders in which a gene defect has a clinically significant impact on a metabolic pathway, initial discussions point to the importance of the subject and the relevance of conflicts of interest policies for ERN networks. This position statement is intended as a first step to support further discussions, which stretches well beyond our own field.

### Objectives

All RD ERNs will be required to deliver added value in at least three of the objectives listed in Article 12 of the Directive on patients’ rights in cross-border healthcare. In summary these objectives are:

- To help realise the potential of European cooperation regarding highly specialised healthcare for patients and for healthcare systems by exploiting innovations in medical science and in health technologies;
- To contribute to the sharing of knowledge regarding sickness prevention;
- To facilitate improvements in diagnosis and the delivery of high-quality, accessible and cost-effective healthcare for all patients with a particular medical condition for which expertise and specialized healthcare facilities are concentrated and only available at a limited number of locations;
- To maximise the cost-effective use of available resources by concentrating them where appropriate;
- To reinforce research, epidemiological surveillance like registries and provide training for health professionals;
- To facilitate the mobility of expertise, virtually or physically, and to develop, share and spread information, knowledge and best practice and to foster developments of the diagnosis and treatment of rare diseases, within and outside the reference networks;
- To encourage the development of quality and safety benchmarks and to help develop and spread best practice within and outside the network;
- To help member states with a low number of patients with a particular medical condition or lacking technology or expertise to provide highly specialised services of high quality.

### Funding

The financial support allowing the individual CEs and affiliated centres to deliver healthcare is the responsibility of the EU member states. However, the set-up and maintenance of ERNs will involve costs for networking activities involved in the set-up of clinical guidelines and protocols including care pathways, development of consensus guidelines and awareness studies, which is currently not covered. According to the recommendations of the EUCERD [5, 6], such network activities should be part of a sustainable funding support mechanism provided from EC funds. Currently, however, it is still unclear what funding for ERN networking will be available and how it will be raised and allocated. For ERNs to work independently, public funding is of crucial importance.

### Role of healthcare professionals, patient organizations and industry

#### Role of healthcare professionals

ERNs are networks of CEs providing healthcare for patients suffering from rare diseases: healthcare providers and the professionals working in such organizations will lead the networks. ERNs will not only need to collaborate with CEs and each other, but also with patient groups, social care providers, and affiliated research groups and diagnostic laboratories. ERNs should therefore have a robust and clearly defined governance with oversight structures and comprehensive methods for evaluation. The coordinating centre of an ERN will need to have and further develop its capability to coordinate the network, and this competence should be regularly evaluated as well.

#### Role of patient organizations

CEs should collaborate with patient organizations to bring in the patient perspective. In addition, patient organizations should play a role in ERNs and their evaluation. There is an on-going discussion that the role of patients and patient organizations should be more robust and better defined at all levels. In several member states, patient organizations are increasingly involved in development of care pathways and research, such as in the French National Plan for Rare Diseases (Plan National Maladies Rares [10] and the Italian National Plan for Rare Diseases [11]). Experiences from these platforms can be used to strengthen the role of patients and their organizations in ERNs.

#### Role of industry

As ERNs primarily engage in clinical activities including the development of clinical guidelines and protocols such as diagnostic and therapeutic care pathways, there should be no role for industry in the governance of ERNs. Yet, for the discovery and development of new medications and medical devices that improve the prevention, diagnosis, and treatment of health problems, research partnerships between industry, academia, public hospitals and government are essential. Such partnerships should be regulated in such a way that principles of professional autonomy of healthcare providers and scientific integrity of researchers are preserved and conflicts of interests are avoided for the ERN as such and in the member CEs. To prevent such conflicts of interest and to prudently handle situations in which they are at stake is not only in the interest of the ERNs, healthcare professionals and patients, but also in the interest of industry. For that reason we propose to adopt basic principles of independence for ERNs and CEs.

## Proposal for a conflict of interest policy

Many healthcare professionals have relationships with industry. The increasing role of patient organizations in all aspects of patient care has also enhanced their interactions with industry. Many of these relationships concern the clinical development of innovative treatments, but industry also supports disease awareness activities, diagnostic recommendations and management guidelines. Even when industry support for such initiatives remains solely financial, the relationships involved carry risks of conflict of interest and undue influence. These kinds of risks, which are well recognised for individual healthcare professionals and institutions or organizations, will also apply to ERNs. A conflict of interest policy is therefore needed with the aim of maintaining the scientific integrity and professional independence of ERNs and CEs. Such a policy usually includes i) the disclosure of financial relationships, ii) the prohibition of certain relationships, and iii) the management of potential conflicts of interest that have been identified [12]. In the paragraphs below, we will first give a short description of the concept of conflicts of interest, followed by an initial proposal for a conflict of interest policy for ERNs, their participating CEs and the individual healthcare professionals involved.

### Conflicts of interest

According to the report of the USA Institute of Medicine (IOM) Committee on Conflict of Interest in Medical Research, Education, and Practice a conflict of interest is a set of circumstances that creates a risk that the professional judgment or actions regarding a primary interest (i.e. promoting and protecting the integrity of research, the welfare of patients, and the quality of medical education) will be unduly influenced by a secondary interest (i.e. financial interests). Secondary interests are unwanted only if they are more important than the primary interest in professional decision-making [12]. Conflict of interest policies aim to safeguard that primary interests guide professional decisions, and not secondary interests. Such policies have proven to work best when they are preventive and corrective rather than punitive [12]. Also, the likelihood of undue influence should be taken into account when developing a conflict of interest policy [12]. At the level of the individual healthcare professional, it is assumed that the probability increases when the value of the secondary interest is greater. Shareholding (or share options) in a pharmaceutical company, for example, creates a great risk for a conflict of interest, since positive results of a clinical trial may directly benefit the healthcare professional involved. Likewise, large fees for serving on a company advisory board carry a greater risk than small honoraria. Other aspects of the relationship, such as its depth and duration, may unduly influence professional decisions; the longer and closer the relationships, the higher the risk. Thirdly, whether the payments are transferred to the personal bank account of the healthcare professional or to the institution’s account is of importance when assessing the risk of undue influence [12]. Relationships with industry and conflicts of interest may not only exist at the individual level, but also at the institutional level and for patient organizations. For example, institutions may rely on industry funding for the appointment of research personnel, the development of treatment guidelines, or other activities. Similar risks may be greater for ERNs as, in the rare disease field, experts are involved in the development of innovative therapies and thus almost invariably have relationships with industry. Going forward, strategies should be developed which will avoid all potential conflicts of interest, but in the first instance, this may be an unrealistic goal. Taking all these things into consideration we propose the following recommendations.

### Recommendations

Principles and regulations to protect scientific integrity and professional independence should be specified for (a) the governance of ERNs including the activities carried out by the ERN; (b) the CEs that will be its members and their activities; (c) external collaborative relations of ERNs and CEs with third parties:

- *ERN governance and activities*
  Costs for networking should be funded by the EC or scientific organizations. Direct funding by industry should be prohibited.
  ERNs should create a strong governance structure, with a steering committee, an independent board of trustees and transparent procedures to avoid and handle potential conflicts of interest and the handling of complaints
  The board of trustees (adapted from [12])
    has no members who themselves have serious conflicts of interest (see below) relevant to the activities of the ERN;
    has no members who are directly in charge of running a CE that is part of the ERN;
    has at least two patient representatives and a government/EC representative with relevant expertise;
    creates adequate and independent arrangements for the day-to-day oversight and the management of institutional conflicts of interest;
    submits an annual report to the steering committee, which should be made public.
  Activities within ERNs, specifically the development of clinical guidelines and protocols such as care pathways, treatment guidelines and diagnostic strategies should
    follow state of the art methodology, including the conduct of systematic and up to date reviews of the evidence and the linking of recommendations to that evidence
    include members that meet the criteria as listed in Table 1 [12]
- * Centres of Expertise and their members*
  CEs wishing to join an ERN should disclose their financial relationships with industry, which will be assessed against the criteria as delineated in Table 2
  CEs with serious conflicts of interest according to the criteria in Table 2 cannot be part of an ERN
  Healthcare professionals involved in CEs and ERNs should publicly disclose their financial relationships with industry
  Healthcare professionals involved in CEs and ERNs with serious conflicts of interest according to the criteria in Table 2 cannot participate in the development of clinical guidelines and protocols such as care pathways, treatment guidelines and diagnostic strategies
- * External collaborative relations with third parties*
  Public-private partnership in the context of ERNs should be possible for the stimulation of innovation, in particular development of new treatments or medical devices and tools that ultimately offers benefit to patient care. The industry is a very valuable partner in this respect. It is expected that ERNs will engage with industry in future applications for Horizon 2020 research grants. These partnerships should be established in such a way that the principles of independence of an ERN and the CEs are maintained.
  The industry cannot be involved in development of clinical guidelines and protocols, such as care pathways, treatment guidelines and diagnostic strategies, as indicated above, nor can they be members of the ERN, steering committee or board of trustees.

**Table 1:** Proposed criteria for participation in the assessment and approval of clinical guidelines and protocols for rare conditions/diseases (adapted from recommendation 7.1 in [12])

Panels and groups that formulate and assess ERN guidelines for clinical practice should generally exclude as panel members individuals with conflicts of interest (see Table 2) and should not accept direct funding from medical product companies or company foundations. Such groups should publicly disclose with each guideline their conflict of interest policies and procedures and the sources and amounts of indirect or direct funding received for development of the guideline. In the situation in which avoidance of panel members with conflicts of interest is impossible because of the critical need for their expertise, then groups should:
• Publicly document that they made a good-faith effort to find experts without conflicts of interest by issuing a public call for members and other recruitment measures;
• Appoint a chair without a conflict of interest;
• Limit members with conflicting interests to an agreed part of the panel;
• Exclude individuals with serious conflicts of interest (see Table 2);
• P3ublicly disclose the relevant conflicts of interest of panel members.

**Table 2:** Proposed criteria for conflicts of interest

1. CEs that are members of an ERN can receive unrestricted grants as well as research grants from a pharmaceutical company provided full and timely (i.e., advance) disclosure of receiving such grants is given to the ERN’s Steering Committee and the amount does not conflict with other criteria
2. Centres of Expertise (CEs) cannot become a member of an ERN if it is primarily funded by and financially dependent on industry for a substantial part (to be defined by an independent body)
3. Healthcare professionals and patient representatives involved in CEs and ERNs cannot take part in the development of clinical guidelines and protocols, such as care pathways, treatment guidelines and diagnostic strategies, if they have the following serious conflicts of interest relevant to the CE or ERN:
a. being employed by a company with commercial interest
b. having equity or other ownership interests in a company with commercial interest
c. receiving fees for work related activities on a personal bank account
d. receiving fees that are disproportional to the work done (i.e., maximum fee more than locally agreed standards per hour)
4. Healthcare professionals and patient representatives involved in CEs and ERNs can take part in the development of clinical guidelines and protocols, such as care pathways, treatment guidelines and diagnostic strategies, if they have the following conflicts of interest, provided that they fully disclose these relationships and compensation is reasonable (according to local standards):
a. performing activities in the context of clinical trials
b. performing consultancies for a company
c. giving presentations during meetings organized by a company
d. receiving reasonable reimbursement of travel and hotel costs as part of meetings organized by a pharmaceutical company

## Summary

A conflict of interest policy with respect to European Reference Networks, Centres of Expertise, individual healthcare professionals and patient organizations is needed to maintain scientific integrity and independence of ERNs. We propose criteria for a transparent structure, which could be used as a guideline for the future set-up of ERNs. The main elements are [1] a strong governance, with a Board of Trustees, whose task will be to monitor the activities and make sure that independence is maintained, and [2] a clearly defined set of criteria for CEs and their members. ERNs should seek endorsement from existing independent working groups. After the ERNs have been established, they can interact with industry for clearly described projects focusing on basic research. To allow for an independent set-up of ERNs, a strategy for public funding should be developed by the EC as soon as possible.
